# Urine Trouble: Forniceal Rupture in the Setting of a Small Urolithic Stone

**DOI:** 10.7759/cureus.112904

**Published:** 2026-07-18

**Authors:** Nicholas D Luke, George Mathew, Mehrdad Alaie

**Affiliations:** 1 Emergency Medicine, St. Barnabas Hospital, Bronx, USA

**Keywords:** acute kidney injury, atraumatic, renal calyceal rupture, urine extravasation, urolithiasis

## Abstract

Fornical ruptures, also known as calyceal ruptures, are a rare sequela of obstructive uropathy in most cases. This pathology can present with similar symptoms to urolithiasis, such as costovertebral tenderness, abdominal pain, nausea, and vomiting. Treatment of forniceal ruptures can vary based on the individual clinical presentation. It may be conservative with hydration, intravenous antibiotics, and pain control. It can also involve more invasive interventions such as stent placement, nephrostomy tube placement, and fluid aspiration. We present a patient with no history of urolithiasis, trauma, malignancy, or previous urologic interventions, who was found to have an obstructive uropathy by a three-millimeter stone that led to a right-sided forniceal rupture by computed tomography imaging. The patient was admitted to the general medical service with urology following for intravenous antibiotics, stent placement with cystoscopy, and lithotripsy.

## Introduction

Urolithiasis is a common pathology involving the obstruction of the urinary system with stones made of various compositions (calcium oxalate or phosphate being the most common) that can account for millions of emergency department visits annually [1]. These stones form within the kidneys and may travel down into the ureter and cause obstructions [1]. In most cases, these obstructing stones can be managed conservatively with pain medications, hydration, diet modification, and medical expulsive therapy such as alpha-blockers. If the stone is large in diameter, it has a lower likelihood of passing spontaneously. A three-millimeter stone may have a passage rate of 76%, while a seven-millimeter stone has a passage rate of 48% [1]. Although larger stones are generally more likely to produce obstruction, even small distal ureteral stones may generate sufficient intrapelvic pressure to produce hydronephrosis and, rarely, forniceal rupture. Forniceal rupture is a rare sequela of obstructive uropathy that leads to extravasation of urine into the extraperitoneal space, the peritoneal cavity, or both [2]. Although the exact incidence rate is not well established due to its rarity, forniceal rupture may drastically alter a patient's clinical course once diagnosed. Contrast-enhanced computed tomography with delayed excretory-phase imaging is an effective modality for diagnosing forniceal rupture and assessing the extent of extravasation with delayed imaging. The extravasation of urine can form urinomas, which may lead to abscess formation, urosepsis, loss of kidney function, and death [2]. Even though a small ureteric stone is expected to most likely pass spontaneously in the majority of cases, it is possible that it may cause obstruction and lead to rupture of the collecting system. We present a case of forniceal rupture in a patient with a three-millimeter stone and without any prior history of urolithiasis or urologic intervention. This case highlights the importance of considering forniceal rupture even in cases of small distal ureteric stones and how computed tomography imaging can promptly diagnose and stratify the severity of obstructive pathology.

## Case presentation

A 65-year-old male with a past medical history of hypertension, type two diabetes mellitus, sciatica, and opioid use disorder presented to the emergency room with one day of nausea, non-bloody and non-bilious vomiting, and right-sided sudden-onset abdominal pain radiating to the right flank. The patient described the pain as colicky and crampy in nature. He denied ever having these symptoms in the past, denied any trauma, recent surgeries, dysuria, hematuria, recent travel, and exposure to sick contacts. 

The patient was hemodynamically stable and afebrile during his hospital visit. His initial vital signs were a heart rate of 103 beats per minute, a blood pressure of 151/78 mmHg, a temperature of 37.3 °C (99.1 °F), a respiratory rate of 19 breaths per minute, and an oxygen saturation of 100% on room air. His initial physical exam was remarkable for right-sided costovertebral tenderness and tenderness in the right lower quadrant. There was no rebound or guarding, and the patient had a negative Murphy's sign and McBurney’s point tenderness. 

**Figure 1:**
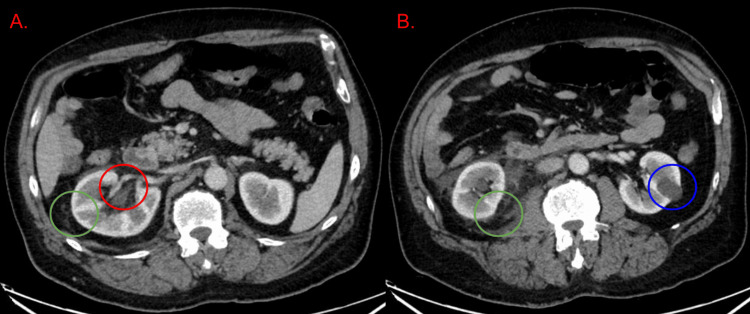
Transverse view from a computed tomography scan with contrast of the abdomen and pelvis A transverse view from a computed tomography scan with contrast of the abdomen and pelvis that depicts mild right-sided hydronephrosis and hydroureter (red circle) on panel A. It also shows right-sided perinephric stranding as well as a moderate amount of perinephric fluid (green circles) on panel A; these findings are nonspecific for forniceal rupture. A renal cyst (blue circle) can be seen on the left kidney on panel B.

**Figure 2:**
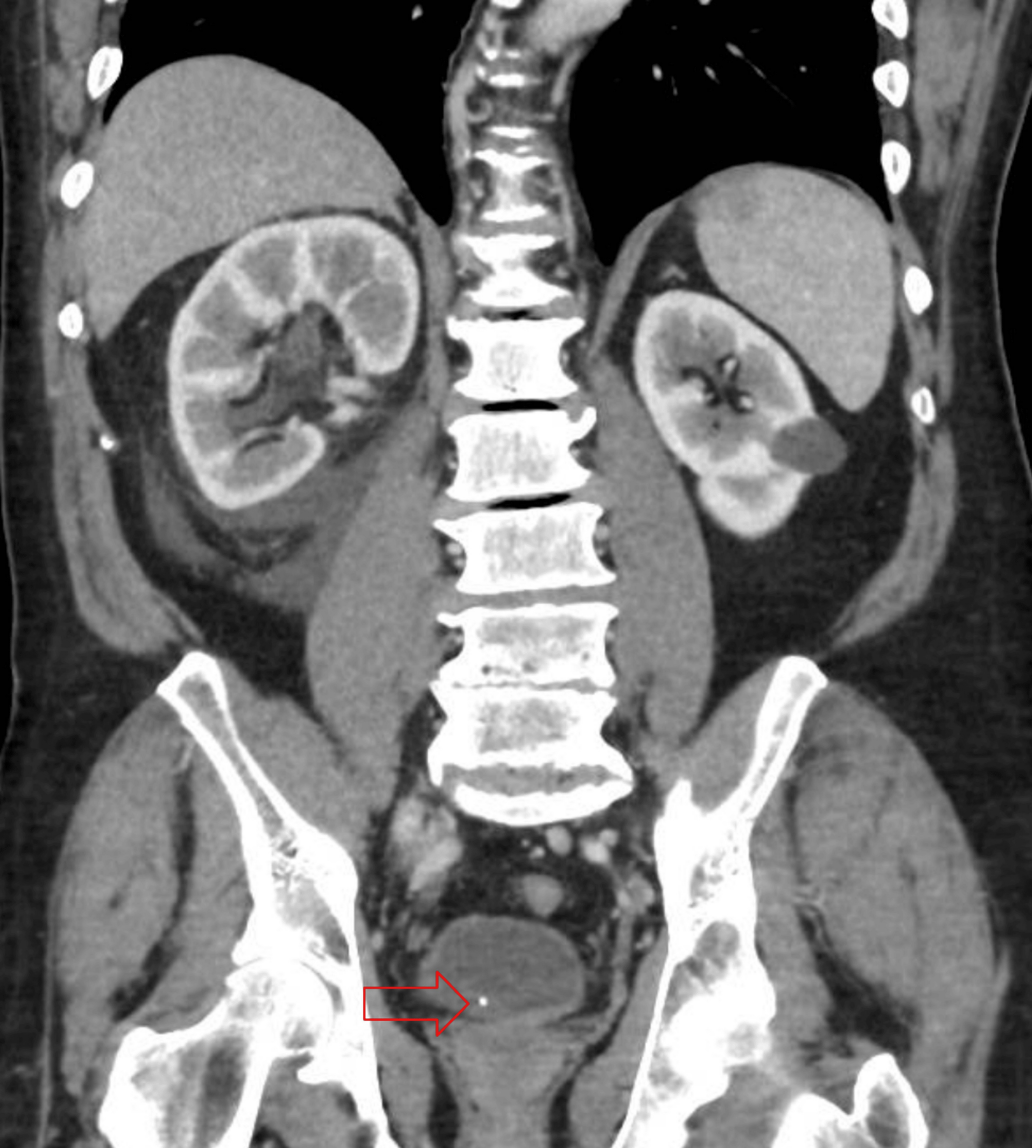
A coronal view depicting a stone measuring 3 × 2 × 2 mm visualized in the right ureterovesical junction (red arrow) on the post-contrast computed tomography scan.

Initially, blood work, a urinalysis, a urine culture, and a computed tomography scan of the abdomen and pelvis were obtained with contrast. The contrast study depicted mild right-sided hydronephrosis and hydroureter (Figure 1). A 3 × 2 × 2 mm stone was seen in the right ureterovesical junction (Figure 2). A moderate amount of right-sided perinephric stranding and perinephric fluid can be seen, extending distally along the right ureter (Figure 1). A left renal cyst was noted as well (Figure 1). The left-sided kidney and ureter otherwise appear normal on the imaging study. The radiologist also recommended a delayed post-contrast computed tomography scan in order to assess for extravasation of the contrast media from the renal collecting system. This repeat post-contrast scan was performed approximately two hours after the initial scan. 

**Figure 3:**
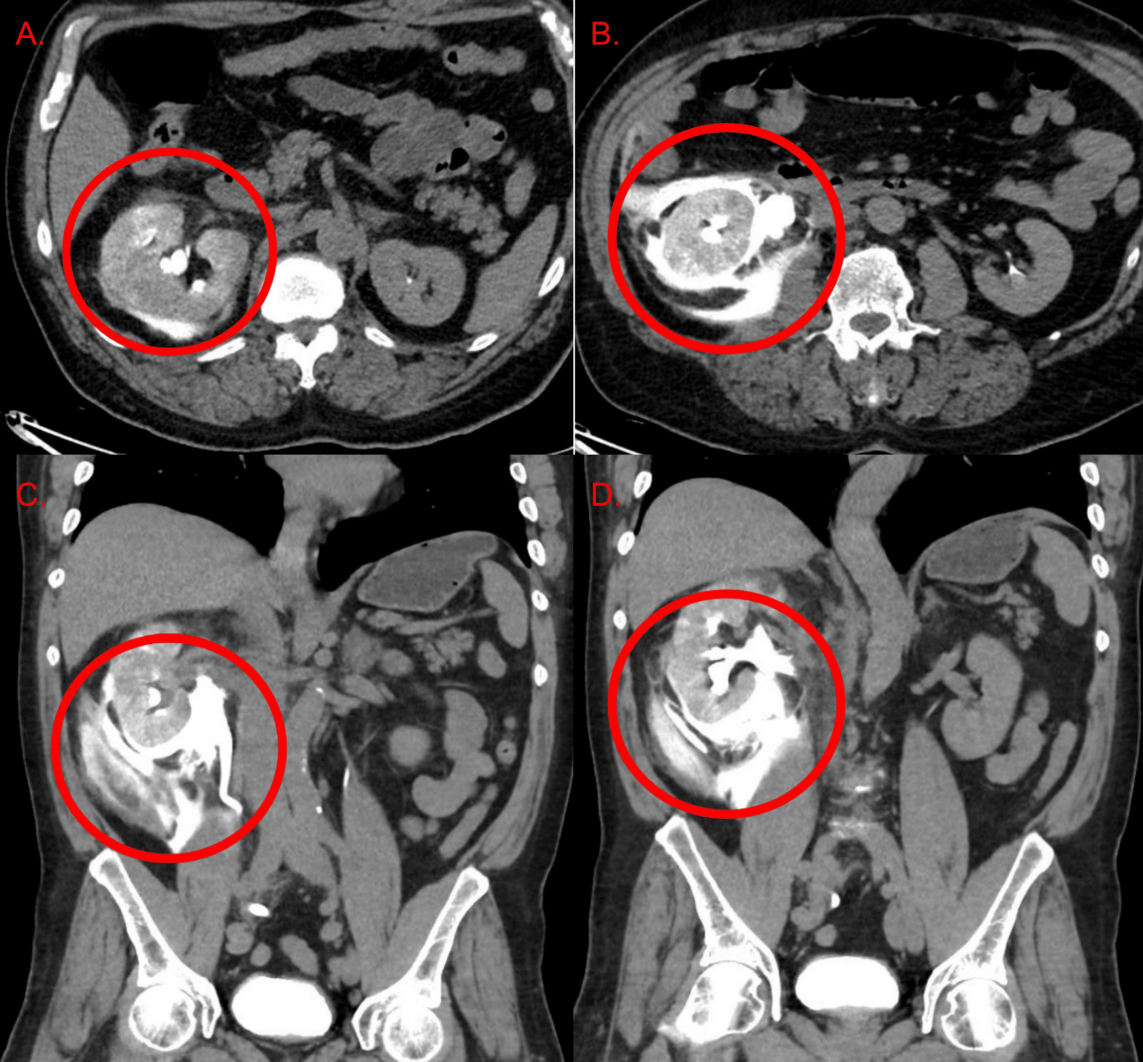
Panels A-D demonstrate the extravasation of contrast media on the right-sided renal collecting system on a delayed, non-contrast computed tomography scan (red circles). This figure demonstrates the importance of delayed imaging in the setting of forniceal rupture because it allows the contrast media sufficient time to be filtered by the kidney and subsequently travel into the collecting system to depict the location and extent of the urinary extravasation.

As the medical workup progressed, a second computed tomography scan was performed, but without contrast this time. The delayed post-contrast study redemonstrated right-sided hydroureteronephrosis without clear visualization of the stone due to contrast in the bladder (Figure 3). It also depicted extravasated contrast surrounding the right kidney and proximal ureter, which is concerning for forniceal rupture (Figure 3). 

The patient’s blood work was remarkable for an elevated white blood cell count, creatinine, and venous lactate (Table 1). The white blood cell count peaked at 15.0 × 10³/µL, and downtrended to 7.4 10*3/uL four days later before discharge. The patient’s creatinine peaked at 2.0 mg/dL (his baseline creatinine was 0.9 mg/dL about three months before this hospital visit). Finally, his lactate rose to 3.0 mmol/L on his initial venous blood gas, and later downtrended to 1.6 mmol/L on his second day of the hospital visit. The urine culture had no growth after 48 hours, and the urinalysis was negative for nitrites, ketones, leukocytes, and blood. The remainder of his laboratory findings were within normal limits.

**Table 1:** Pertinent positive laboratory findings seen in our patient upon initial presentation to the emergency room, with reference ranges.

Clinically Significant Markers	Abnormal Laboratory Results	Reference Range
Creatinine	2.0	0.7 - 1.3 mg/dL
White Blood Cell Count	15.0	4.5 x 10^3 - 11 x 10^3 / mcL
Venous Lactate	3.0	0.5 - 2.0 mmol/L

The patient was evaluated by the urology service, which recommended the initiation of daily tamsulosin at 0.4 mg to facilitate spontaneous stone passage, starting broad-spectrum antibiotics (vancomycin and ceftriaxone were chosen), pain control with antiemetics, oral and/or intravenous hydration, and stent placement via cystoscopy with laser lithotripsy. The patient was admitted to the general medical floor for intravenous antibiotics, with urology closely following. The patient was offered intravenous antibiotics due to the urinary extravasation secondary to the forniceal rupture. Based on the elevated white blood cell count and venous lactate, there was a concern for urosepsis and abscess formation. So the utilization of antibiotics was justified based on this, rather than the negative urinalysis, urine culture, and blood culture. The patient was offered stent placement via cystoscopy with laser lithotripsy; however, he declined it during his hospital stay, so no stone analysis was obtained. No further imaging was performed during his hospital visit. The absence of follow-up imaging prevented radiologic confirmation of resolution of the extravasation and hydronephrosis. He was discharged after staying for four days in the hospital with resolution of symptoms, was prescribed tamsulosin, was given outpatient urology follow-up, and his kidney function had returned to baseline (0.8 mg/dL). Discharge without decompression of the rupture was appropriate due to the improvement in kidney function, resolved lactate, and lack of tenderness on physical exam.

## Discussion

A renal forniceal rupture is a rare sequela of obstructive uropathy. Since not all stones cause obstruction, it is not commonly seen. However, if the pressure within the renal collecting system becomes too high, then the delicate fornix is prone to tears and rupture. In some cases, certain patient demographics are more likely to get forniceal ruptures secondary to obstructive uropathy. One such demographic is pregnancy. In one example, Upputalla et al. report a case of a 26-year-old female at twenty-three weeks of gestation who experienced a right-sided forniceal rupture without any signs of obstructive uropathy [3]. Hormonal changes associated with pregnancy most likely led to the rupture of the renal fornix. These changes include structural and endocrinological factors. The kidneys increase in size by one to two centimeters due to an increase in the renal vascular volume, which will also increase the glomerular filtration rate by 50% [3].

There is a certain amount of physiologic hydronephrosis and hydroureter that occurs due to compression by the uterus as it expands during pregnancy [3]. Another physiologic change during pregnancy that may affect the risk of forniceal rupture is the increase in pelvic vasculature. The increase in blood flow to the pelvis from the iliac and ovarian vessels compresses the ureter, more often on the right than on the left side [3]. From an endocrinological standpoint, progesterone and prostaglandins can lead to a decrease in ureteral tone and peristalsis [3]. These factors can all lead to increased pressure within the renal collecting system and increase the chances of spontaneous forniceal rupture. Besides pregnancy, other risk factors that can lead to an increased risk of forniceal rupture include anatomical variants such as horseshoe kidneys or ureteral strictures, or compression by tumors [4]. Unlike many previously published reports involving pregnancy or anatomic abnormalities, our patient had no prior urologic disease and developed rupture from a relatively small distal ureteral stone.

The underlying mechanism of forniceal rupture in our patient is not completely clear. Hypothetically, it is possible that it may have been multifaceted. Perhaps a delay in seeking medical attention may have led to the rupture, which raises the question: If our presenting patient had come to the emergency room within hours of symptom onset versus a day later, would the chance of a forniceal rupture have decreased? The clinical suspicion for anatomical abnormalities is low in our patient due to no anatomical variations noted on two different imaging studies.

The management of a forniceal rupture largely depends on the clinical presentation of the patient. The clinical presentation can dictate whether the patient will receive more conservative management with pain control and intravenous antibiotics or will require more invasive treatments such as stent placement or fluid aspiration. Our presenting patient had an acute kidney injury, was hemodynamically stable, and had no previous history of urologic intervention or trauma. Since our presenting patient declined lithotripsy and stent placement, more conservative management with intravenous antibiotics, hydration, symptom control, and elective stent placement via cystoscopy with lithotripsy. 

On the more invasive side of management, Al-Mujalhem et al. studied the indications of conservative management versus the utilization of invasive procedures to treat forniceal ruptures [5]. Al-Mujalhem et al. were able to manage about 57.5% of their patients in the study via conservative therapy [5]. The remainder of the cases required some form of interventional management based on persistent pain, fever, acute kidney injury, urinoma size, and/or solitary kidney [5]. Such interventions included double-J stent insertions, nephrostomy tube insertions, ureteroscopy, and urinoma drainage if the volume exceeded 100 milliliters [5]. In our presenting patient, conservative management was ultimately utilized because the patient was hemodynamically stable and had a resolution of his venous lactate and creatinine after fluid administration.

The clinical severity of a forniceal rupture can vary greatly based on the initial presentation. However, the sequelae of the rupture can have a lasting impact on the patient and thus require prompt treatment. During obstructive uropathy, the excess pressure within the renal collecting system can potentially exceed the tensile strength of the calyceal walls [6]. Once the tensile strength is overcome, a rupture is imminent and can further complicate a clinical course once a leakage of urine starts to occur. Without a timely diagnosis and proper management, worsening complications from a forniceal rupture can include urosepsis, perinephric abscess, retroperitoneal fibrosis, loss of renal function, and even death [6]. Timely management may also prevent the need for more aggressive and invasive surgical intervention before a forniceal rupture may occur in the setting of obstructive uropathy. Interventions such as stents and fluid drainage, which also come with their own risks. While forniceal ruptures are quite rare, with no exact documentation of their incidence, the pathology and its possible sequelae underscore the need for early diagnosis and treatment to avoid future hospitalizations and promote beneficial outcomes [6].

## Conclusions

Obstructive uropathy is a common reason for hospital visits; however, it may result in a rare and unforeseen complication known as forniceal rupture. Forniceal ruptures should be diagnosed and treated promptly to minimize the risk of urosepsis, abscess formation, and the need for invasive management such as stent placement and retroperitoneal fluid aspiration. While there are limitations to drawing broad conclusions from a single case presentation, clinicians should maintain suspicion for forniceal rupture in patients with persistent or severe symptoms, regardless of stone size. Prompt recognition may facilitate appropriate monitoring and treatment, as complication prevention cannot be established from a single case. A forniceal rupture may present on a wide clinical spectrum, from mild symptoms to life-threatening urosepsis. Further studies or case accumulation (such as multicenter observational studies, registries, or larger case series) may facilitate the understanding of the incidence and risk factors for forniceal rupture, and help shape an algorithmic management pathway for treating and stratifying forniceal rupture.
